# Convergence of Subjective Outcome and Objective Outcome Evaluation Findings: Insights Based on the Project P.A.T.H.S.

**DOI:** 10.1100/tsw.2007.59

**Published:** 2007-02-19

**Authors:** Daniel T. L. Shek, Tak Yan Lee, Andrew M. H. Siu, Hing Keung Ma

**Affiliations:** ^1^ Quality of Life Centre, Hong Kong Institute of Asia-Pacific Studies, The Chinese University of Hong Kong, Shatin, Hong Kong; ^2^ Social Welfare Practice and Research Centre, Department of Social Work, The Chinese University of Hong Kong, Shatin, Hong Kong; ^3^ Department of Applied Social Studies, City University of Hong Kong, Hong Kong; ^4^ Department of Rehabilitation Sciences, Hong Kong Polytechnic University, Hong Kong; ^5^ Department of Education Studies, Hong Kong Baptist University, Hong Kong

**Keywords:** Subjective outcome evaluation, objective outcome evaluation, client satisfaction approach, Chinese, adolescents

## Abstract

A total of 546 students participated in the Tier 1 Program of the P.A.T.H.S. Project responded to the Chinese Positive Youth Development Scale (CPYDS) at pretest and posttest and the Subjective Outcome Scale (SOS) at posttest. Result showed that the SOS was internally consistent. The SOS total scores were significantly related to measures of global satisfaction and the participants' degree of sharing with others, thus giving support to its construct validity. Factor analysis revealed that there were three dimensions of the scale and the related subscales were significantly correlated among themselves. Based on the significant relationships between the SOS measures of perceived program effectiveness and posttest CPYDS scores as well as changes in CPYDS scores, the present study revealed the convergence of subjective outcome evaluation findings and objective outcome evaluation findings in the P.A.T.H.S. Project.

## INTRODUCTION

Subjective outcome evaluation or the client satisfaction approach is a popular approach utilized by human service professionals to assess the perceptions of the clients regarding the program, including its format, implementation process, workers and benefits. Despite its popular usage in different service contexts, including the fields of education and social welfare, there are many criticisms against the use of this approach in program evaluation. For example, Weinbach[1] argued that there are several problems of client satisfaction surveys. First, the clients may not tell the truth, thus creating biases in the findings. Second, as those who return the feedback forms are those who stay until the end of the program, there will be completion bias involved. In addition, it would be difficult to obtain random samples based on the program participants. Third, as the respondents may feel grateful or pleased to be asked, bias may be introduced by "I appreciate your asking" phenomenon. Fourth, the generally favorable results based on the subjective outcome evaluation approach may constitute positive bias. Finally, there is the common illusion that client satisfaction equals to successful intervention. Regarding the last problem, Weinbach[1] explicitly warned that "the major problem of using client-satisfaction surveys as indicators of intervention effectiveness, or of quality of a service, is that satisfaction with services and successful intervention are not the same" (p.38). According to Royse[2], the lack of standardized assessment tools for conducting client satisfaction survey also introduces biases for the client satisfaction approach. As such, he recommended the use of assessment tool with known reliability and validity, which would "eliminate many of the problems found in hastily designed questionnaires" (p. 265).

The criticism that perceived effectiveness by the client may not correspond to positive change in objective outcomes definitely appears to be intuitively appealing. Surprisingly, a survey of the literature shows that except in the health-related fields, very few researchers have examined the degree of convergence of subjective outcome evaluation findings and objective outcome evaluation findings in the fields of education and social welfare. Among the published studies, the research findings in the literature are equivocal. For example, while LaSala[3] reported that "client satisfaction was found to be correlated with client report of improved ability to handle problems, whether clients would recommend services to others, whether the fee was considered fair, and client and therapist ratings of global improvement" (p.54), Walsh and Lord[4] commented that "a small number of studies have used other outcome measures and failed to find a strong relationship between higher client satisfaction and other indicators of successful intervention" (p. 41). Furthermore, literature shows that no scientific studies have been published in the Chinese context on subjective outcome evaluation tools and the relationship between subjective outcome and objective outcome findings.

Besides the limited number of studies investigating the relationship between subjective and objective outcome measures, there are many methodological problems intrinsic to the existing studies. For example, the sample size in the existing studies was usually small. In the study conducted by LaSala[3], only 100 clients were recruited. In the study by Walsh and Lord[4], data based on only 19 parents were used to support the claim that there was no relationship between objective outcome measure and subjective outcome measure. Obviously, such small sample size substantially reduces the power of the statistical analyses. Furthermore, few researchers have examined the relationship between subjective outcome and objective outcome evaluation findings using multiple indicators.

The Project P.A.T.H.S. (Positive Adolescent Training through Holistic Social Programmes) is a multi-year universal positive youth development program to promote holistic adolescent development in Hong Kong, which is financially sponsored by the Hong Kong Jockey Club Charities Trust[5,6]. There are two implementation phases in this project – Experimental Implementation Phase and Full Implementation Phase. For the Experimental Implementation Phase (January 2006 to August 2006), 52 secondary schools participated in the project with the objectives of accumulating experience in program implementation and familiarizing frontline workers with the program design and philosophy. Evaluation findings based on different evaluation mechanism have been collected to examine the program effects in the Experimental Implementation Phase[7,8,9,10].

To examine the program effect of the Experimental Implementation Phase (2005-06), a quasi-experimental design was utilized[7]. At pretest and posttest, the participants responded to the Chinese Positive Youth Development Scale (CPYDS[11,12]). In addition, the participants responded to the Subjective Outcome Scale (SOS) at posttest. There are 15 items in the SOS that assess the participant's evaluation of different domains, including the program implementation process, instructor and perceived effectiveness.

As both objective and subjective outcome evaluation findings were collected in the above-mentioned study[7], it would be important to ask how the objective outcome evaluation findings are related to the subjective outcome evaluation findings. There are two purposes of this paper. First, the psychometric properties of the Chinese Subjective Outcome Scale (SOS) are examined. In particular, its internal consistency and construct validity would be examined. As far as construct validity is concerned, it is expected that the SOS scores would be related to measures of global satisfaction with the program and the extent to which the participants shared their program experiences with others. In addition, it would be expected that the three dimensions in the scale (i.e., quality of program implementation, quality of instructor and perceived effectiveness) would be correlated among themselves. The second objective of the study is to report some initial findings on the relationship between objective outcome and subjective outcome findings.

## METHODS

### Participants and Procedures

Among the 52 schools joining the experimental implementation phase, there were 29 schools adopting the full program (i.e., 20-hour program involving 40 units). To allow for a longer duration of the program implementation, 14 schools implemented the program before the official implementation date (i.e., January 1, 2006). Among the 7 schools that began the implementation of the program in November to December 2005, four schools were randomly selected to participate in this study. There were 656 and 652 students participated in the pretest and posttest, respectively. Discarding those questionnaires that showed doubtful responses (19 questionnaires at pretest and 22 questionnaires at posttest), usable questionnaires at pretest and posttest could be successfully matched for 546 students.

At pretest and posttest, the purpose of the study was mentioned, and the confidentiality of the data collected was repeatedly emphasized to all of the students in attendance on the day of testing. The students were asked to indicate their wish if they did not want to participate in the study at the time of administration at each wave of data collection (i.e., "passive" informed consent was obtained from the students). All participants responded to all scales in the questionnaire in a self-administration format. Adequate time was provided for the participants to complete the questionnaire.

### Instruments

At pretest and posttest, the participants were invited to respond to a questionnaire including measures of positive youth development, thriving, life satisfaction, adolescent problem behavior and demographic information. The measures focused in this paper are described below.

### The Chinese Positive Youth Development Scale (CPYDS)

Regarding the assessment of positive development of the program participants, the Chinese Positive Youth Development Scale (CPYDS) was used. The CPYDS was found to possess adequate psychometric properties[11,12]. Based on reliability analyses of both the pretest and posttest data in the present study, there were some slight modifications in the composition of the items of the 15 subscales of the CPYDS as follows:

1. Resilience Subscale (6 items): α =.83 and .82 at pretest and posttest.
2. Social Competence Subscale (7 items): α =.80 and .86 at pretest and posttest.
3. Emotional Competence Subscale (6 items): α =.83 and .86 at pretest and posttest.
4. Cognitive Competence Subscale (6 items): α =.85 and .87 at pretest and posttest.
5. Behavioral Competence Subscale (6 items): α =.70 and .62 at pretest and posttest.
6. Moral Competence Subscale (6 items): α =.76 and .78 at pretest and posttest.
7. Self-Determination Subscale (modified 4 items): α =.82 and .82 at pretest and posttest.
8. Self-Efficacy Subscale (modified 2 items): α =.48 and .54 at pretest and posttest.
9. Beliefs in the Future Subscale (modified 2 items): α =.70 and .66 at pretest and posttest.
10. Clear and Positive Identity Subscale (7 items): α =.84 and .86 at pretest and posttest.

11. Spirituality Subscale (7 items): α =.85 and .88 at pretest and posttest.
12. Bonding Subscale (6 items): α =.83 and .85 at pretest and posttest.
13. Prosocial Involvement Subscale (5 items): α =.80 and .86 at pretest and posttest.
14. Prosocial Norms Subscale (5 items): α =.73 and .78 at pretest and posttest.
15. Recognition for Positive Behavior Subscale (4 items): α =.77 and .79 at pretest and posttest.

According to Shek et al.[11,12], the total score based on all 15 subscales could be used as an overall measure of positive youth development. Besides, other composite indices were also formed[7]. In this paper, the total score based on all 15 subscales was used as an overall measure of positive youth development.

### Subjective Outcome Evaluation

Fifteen items were used to assess the participants' satisfaction with the course and instructor as well as their perceived benefits of the program at posttest. There are three areas of items in the scale: a) perceived program implementation quality (6 items); b) perceived quality of the workers (4 items); and c) perceived effectiveness of the program (5 items). Besides, the participants were asked to respond to three items assessing their global perceptions and satisfaction with the program. Finally, the respondents were asked to respond to two items assessing whether they had shared their experiences with their friends and family members.

## RESULTS

The findings showed that the SOS was internally consistent in the present sample. The values of the corrected item-total correlation, mean inter-item correlation coefficients and coefficient alpha observed were also on the high side. The related findings can be seen in Table 1.

As the items of the SOS were highly correlated, a non-orthogonal factor extraction procedure (alpha factoring) was used to analyze the responses of the participants to the items of the scale, yielding two factors with eigenvalues exceeding unity, explaining 74.06% of the variance. As eigenvalue is just one factor to be considered in determining the number of factors[13] and there is conceptual justification to consider the third factor, three factors were extracted even though the third factor did not have an eigenvalue exceeding unity. The three-factor solution was then rotated to a promax criterion for interpretation. Factor I included items 1, 2, 3, 4, 5 and 6 which was labeled Quality of Program Implementation (SOS-Process), explaining 64.60% of the variance. The second factor included items 11, 12, 13, 14 and 15, which could be labeled Perceived Effectiveness (SOS-Effect), explaining 9.46% of the variance. Finally, the third factor included items 7, 8, 9 and 10, which was labeled as Quality of Worker (SOS-Worker). Although the eigenvalue of this factor was not high, it explained 4.44% of the variance.

To examine the stability of the factors derived from the SOS, analyses based on coefficients of congruence were computed by randomly splitting the total sample into two sub-samples. Results showed that the related coefficients of congruence were high (coefficients of congruence = .99, .96 and .96 for SOS-Process, SOS-Effect, and SOS-Worker, respectively). The findings on the pattern matrix of the analyses in the three samples can be seen in Table 2.

**TABLE 1**
**Reliability of the Subjective Outcome Scale (SOS)**

| Item | Correlated Item-total Correlation |
|---|---|
| 1. The atmosphere of the class was good. | .76 |
| 2. There were many opportunities for students to exchange ideas during class. | .77 |
| 3. I often had encouragement from the responses of classmates. | .75 |
| 4. I think the students actively participated in the class activities. | .74 |
| 5. I think I actively participated in the class activities. | .71 |
| 6. I think the discipline in class was good. | .62 |
| 7. The instructor(s) could arouse my interest in the course. | .82 |
| 8. The instructor(s) could arouse my learning motivation. | .84 |
| 9. The instructor(s) could promote discussion and participation among the students. | .80 |
| 10. I could get encouragement from the responses of the instructor(s). | .78 |
| 11. I think this course can strengthen my ability to face the challenge of life. | .82 |
| 12. I think this course can strengthen my ability to face adversity. | .80 |
| 13. I think this course can increase my understanding about myself. | .82 |
| 14. I think this course can promote my overall development. | .80 |
| 15. Overall speaking, I think the course is helpful to me. | .75 |

Mean inter-item correlation = .62 Alpha = .96

**TABLE 2**
**Factor pattern matrix (alpha extraction followed by Promax) of the Subjective Outcome Scale (SOS)**

| Item | Total | | | Sub-sample 1 | | | Sub-sample 2 | | |
|---|---|---|---|---|---|---|---|---|---|
| | F1 | F2 | F3 | F1 | F2 | F3 | F1 | F2 | F3 |
| 1 | *.810* | .033 | .021 | *.865* | -.106 | .102 | .175 | *.737* | -.033 |
| 2 | *.859* | -.027 | .055 | *.895* | -.113 | .122 | .068 | *.814* | -.009 |
| 3 | *.836* | .072 | -.052 | *.806* | .143 | -.074 | .036 | *.868* | -.068 |
| 4 | *.806* | -.021 | .056 | *.739* | .146 | .029 | -.151 | *.857* | .120 |
| 5 | *.752* | .041 | .017 | *.731* | .151 | -.065 | .023 | *.763* | .063 |
| 6 | *.646* | .018 | .044 | *.637* | .007 | .099 | .032 | *.617* | .017 |
| 7 | .004 | .128 | *.802* | .036 | .177 | *.718* | .113 | -.030 | *.864* |
| 8 | .034 | .113 | *.805* | .031 | .188 | *.732* | .058 | .038 | *.871* |
| 9 | .137 | .034 | *.732* | .057 | .032 | *.803* | .069 | .245 | *.613* |
| 10 | .052 | .188 | *.634* | .096 | .158 | *.596* | .236 | .046 | *.631* |
| 11 | .073 | *.737* | .104 | .075 | *.680* | .176 | *.765* | .073 | .063 |
| 12 | .037 | *.832* | .027 | .052 | *.723* | .096 | *.871* | .038 | .019 |
| 13 | .080 | *.849* | -.001 | .058 | *.855* | .006 | *.856* | .077 | .005 |
| 14 | .023 | *.934* | -.052 | .049 | *.929* | -.054 | *.935* | -.003 | -.041 |
| 15 | -.088 | *.777* | .150 | -.081 | *.732* | .167 | *.835* | -.109 | .136 |
| Variance Explained (%) | 64.60 | 9.46 | 4.44 | 65.32 | 8.53 | 4.40 | 64.02 | 10.34 | 4.68 |

Note: F1 = Factor 1. F2 = Factor 2. F3 = Factor 3. The highest loading among the factors for an item is in italics.

There are several lines of evidence that provide support for the construct validity of the SOS. Theoretically speaking, it can be argued that perceptions of program implementation process and the worker should be related to perceived effectiveness of the program. Based on this reasoning, results based on the multi-stage Bonferroni correction procedures[14] showed that these two dimensions of the SOS were significantly correlated with SOS-Effect. Furthermore, participants' perceptions of the three domains were related to global satisfaction with the program as a whole. Finally, the three domains were significantly related to the extent that the participants shared the program experiences with others. The Bonferroni-corrected correlation coefficients are shown in Table 3.

**TABLE 3**
**Correlations between the various measures of subjective outcome evaluation**

| | SOS- Process | SOS- Worker | SOS- Effect | GLOBAL | SHARE |
|---|---|---|---|---|---|
| SOS- Process | .92 | | | | |
| SOS- Worker | .73* | .93 | | | |
| SOS- Effect | .68* | .81* | .95 | | |
| GLOBAL | .76* | .85* | .77* | .89 | |
| SHARE | .62* | .68* | .74* | .67* | .87 |

Note. SOS-Process: Mean score of item 1 to item 6 of the Subjective Outcome Scale. SOS-Worker: Mean score of item 7 to item 10 of the Subjective Outcome Scale. SOS-Effect: Mean score of item 11 to item 15 of the Subjective Outcome Scale. GLOBAL: Mean score of the 3 items assessing global evaluation of the program. SHARE: Mean score of the 2 items assessing the degree of sharing with friends and family members. Coefficient alphas for the various measures are shown in the diagonal.

A two-tailed multistage Bonferroni procedure was used to analyze the data in each sample at each time point. *pFW* is based on the familywise Type 1 error rate. *pT* is the Type 1 error rate per test.

* *pFW* < .05; *pT* < .005

**TABLE 4**
**Correlations on the items on perceived effectiveness and objective outcome measures indexed by the CPYDS and its change score**

| Item | | CPYDS | DCPYDS |
|---|---|---|---|
| 11 | I think this course can strengthen my ability to face the challenge of life. | .54* | .25* |
| 12 | I think this course can strengthen my ability to face adversity. | .55* | .24* |
| 13 | I think this course can increase my understanding about myself. | .58* | .30* |
| 14 | I think this course can promote my overall development. | .55* | .25* |
| 15 | Overall speaking, I think the course is helpful to me. | .54* | .25* |

Note. Items 11 to 15: 5 items on the perceived effectiveness of the program. CPYDS: Scale score of the Chinese Positive Youth Development Scale (mean of Resilience, Social Competence, Emotional Competence, Cognitive Competence, Behavioral Competence, Moral Competence, Self-Determination, Self-Efficacy, Spirituality, Beliefs in the Future, Clear and Positive Identity, Recognition for Positive Behavior, Prosocial Involvement, Prosocial Norms, and Bonding). DCPYDS: Difference score by subtracting CPYDS score at pretest from CPYDS score at posttest.

A two-tailed multistage Bonferroni procedure was used to analyze the data in each sample at each time point. *pFW* is based on the familywise Type 1 error rate. *pT* is the Type 1 error rate per test.

* *pFW* < .05; *pT* < .005

Because it is conceptually more important to look at perceived effectiveness of the subjective outcome measures, the items in the SOS-Effect were particularly focused upon in the present paper. To examine the relationship between the subjective outcome measures and objective outcome measures, three sets of analyses were conducted. First, Pearson correlation coefficients on the relationships between the related items and the Chinese Positive Youth Development Scale (CPYDS) at posttest were computed. Results showed that these items were highly related to the posttest CPYDS scores. Second, Pearson correlation coefficients on the relationships between these measures and the difference scores based on the CPYDS (posttest CPYDS score minus pretest CPYDS score) were computed. Results similarly showed that the subjective outcome evaluation scores were significantly correlated with the difference scores. The Bonferroni-corrected correlation coefficients are shown in Table 4.

Because the approach based on difference score may be criticized as unreliable because of variability in the responses of the participants, another approach based on hierarchical multiple regression analyses involving two steps was performed. In Step 1, the effect of Time 1 CPYDS scores on Time 2 CPYDS scores was removed. In Step 2, the effect of a SOS-Effect item on the residualized Time 2 CPYDS scores was assessed. Using this approach, the effect of subjective outcome evaluation item on the change in the CPYDS scores could be examined. As shown in Table 5, the subjective outcome evaluation items had significant predictive effect on CPYDS scores at posttest after controlling the effect of pretest CPYDS scores.

## DISCUSSION

The psychometric properties of the Subjective Outcome Scale (SOS) were examined in this paper. Reliability analyses showed that the SOS designed for the Project P.A.T.H.S. is internally consistent. The present study also provides some support for the construct validity of the subscales in the SOS. Consistent with our predictions, respondents who perceived the program and worker to be more positive generally perceived the program to be more effective. Furthermore, the three measures of domain-specific subjective outcomes were significantly related to measures of global satisfaction and the participant's readiness to share his/her learning experiences with others. Because there are no published studies on subjective outcome evaluation tools in the Chinese literature, the present study can be regarded as pioneering. Because the use of standardized subjective outcome assessment tools can help to enhance the objectivity of client satisfaction survey[2], it is recommended that the psychometric properties of the instruments used in client satisfaction surveys in the welfare and education fields should routinely be examined before they are used in the practice settings on a large scale basis.

Based on factor analyses, it was found that there are three dimensions intrinsic to the SOS: Quality of Program Implementation, Quality of Worker, and Perceived Effectiveness of the Program. Because few research studies have examined the dimensionality of subjective outcome assessment scales in the Chinese context, the present study can be regarded as ground breaking. In addition, the present study demonstrates the importance of performing factor analyses to uncover the dimensions in the related scales and reliability analyses to examine the internal consistency of the dimensions extracted. Nevertheless, as the Eigenvalue of the SOS-Worker factor was low, further studies of the factor structure of the SOS are necessary.

Using the discrete items of the SOS-Effect as measures of perceived effectiveness, results showed that the related measures were associated with the posttest CPYDS scores (with moderate effect size) and the difference score (with low effect size). Furthermore, hierarchical multiple regression analyses showed that the level of perceived effectiveness was actually related to changes in the CPYDS scores. Generally speaking, the findings suggest that a higher level of perceived effectiveness predicted an improvement of CPYDS score at posttest after controlling the effect of CPYDS at pretest. Taken as a whole, the findings clearly suggest that subjective outcome is a measure that is intimately linked to objective outcome indicators. As there are few studies in the field of evaluation on this issue and no related scientific findings have been published in the Chinese context, the present study constitutes interesting and pioneering additions to the literature.

There are three strengths of this study. First, the evaluation findings are based on a large sample size (N=546). Such a big sample size is in sharp contrast to the existing studies in the field in which small sample size was commonly used. Second, different aspects of subjective outcome, including views of the program and the worker as well as perceived effectiveness, were covered in the study. Third, in contrast to the common practice in the evaluation field in the Chinese context, the psychometric properties of the SOS were examined. As the positive responses of the students may be interpreted in terms of their fear of responding in a negative manner, particularly in view of the fact that teachers are commonly regarded as authority figures in the Chinese culture, the present findings provide insight to our understanding of the positive subjective outcome evaluation findings found in the Experimental Implementation Phase of the project[15]. In conjunction with the subjective outcome evaluation findings based on the teachers[16], the present findings reinforce the conclusion that there are positive program effects at both the objective outcome and subjective outcome levels.

**TABLE 5**
**Prediction of changes in objective outcome measures at posttest by subjective outcome measures**

| Dependent Variable | Step 1 | | | Step 2 | | | | | |
|---|---|---|---|---|---|---|---|---|---|
| | Predictor | Beta | $R^2$ | Predictor | Beta | Predictor | Beta | F Change | $\Delta R^2$ |
| Time 2 CPYDS | Time 1 CPYDS | .33* | .11 | Time 1 CPYDS | -.00ns | Item 11 | .55* | 140.16* | .29 |
| Time 2 CPYDS | Time 1 CPYDS | .35* | .12 | Time 1 CPYDS | .01ns | Item 12 | .54* | 138.23* | .30 |
| Time 2 CPYDS | Time 1 CPYDS | .33* | .11 | Time 1 CPYDS | -.06ns | Item 13 | .62* | 191.45* | .34 |
| Time 2 CPYDS | Time 1 CPYDS | .33* | .11 | Time 1 CPYDS | -.01ns | Item 14 | .56* | 147.60* | .30 |
| Time 2 CPYDS | Time 1 CPYDS | .32* | .10 | Time 1 CPYDS | -.02ns | Item 15 | .55* | 140.99* | .29 |

Note. Items 11 to 15: 5 items on the perceived effectiveness of the program. CPYDS: Scale score of the Chinese Positive Youth Development Scale (mean of Resilience, Social Competence, Emotional Competence, Cognitive Competence, Behavioral Competence, Moral Competence, Self-Determination, Self-Efficacy, Spirituality, Beliefs in the Future, Clear and Positive Identity, Recognition for Positive Behavior, Prosocial Involvement, Prosocial Norms, and Bonding subscales).

* $p < .0001$

ns non-significant

On the other hand, it is noteworthy that there are several limitations of the study. First, as the Eigenvalue for the SOS-Worker factor was low, there is a need to further examine this observation in future studies. Second, as the findings were based on 4 schools randomly selected from the participating schools, it would be illuminating if more schools could be recruited to participate in the study. Finally, as the duration between the pretest and the posttest was less than one year, it would be exciting if the relationship between the subjective outcome measures and the changes in the objective outcome measures over a longer period of time could be examined. Despite these limitations, the present findings suggest that the SOS is an objective measure of subjective outcome evaluation and the related scores on the SOS-Effect subscale are intimately linked to objective outcome scores indexed by the Chinese Positive Youth Development Scale.

## ACKNOWLEDGEMENTS

The preparation for this paper and the Project P.A.T.H.S. were financially supported by the Hong Kong Jockey Club Charities Trust.
